# The Study of the Aggregated Pattern of TX100 Micelle by Using Solvent Paramagnetic Relaxation Enhancements

**DOI:** 10.3390/molecules24091649

**Published:** 2019-04-26

**Authors:** Liang Zhang, Xin Chai, Peng Sun, Bin Yuan, Bin Jiang, Xu Zhang, Maili Liu

**Affiliations:** 1 State Key Laboratory of Magnetic Resonance and Atomic and Molecular Physics, National Center for Magnetic Resonance in Wuhan, Wuhan Institute of Physics and Mathematics, Chinese Academy of Sciences, Wuhan 430071, China; liangtxdy@163.com (L.Z.); chaixin20@126.com (X.C.); sunpeng@wipm.ac.cn (P.S.); yuankanxue@163.com (B.Y.); jbin@wipm.ac.cn (B.J.); 2 University of Chinese Academy of Sciences, Beijing 100049, China; 3 School of Physics and Optoelectronic Engineering, Yangtze University, Jingzhou 430023, China

**Keywords:** solvent Paramagnetic Relaxation Enhancement (sPRE), TX100, aggregation pattern

## Abstract

TX100 (Triton X-100) is a typical nonionic surfactant that is widely used in biology. However, the detailed aggregated conformation of TX100, such as the boundary between the polar region and the nonpolar region, and the arrangement of hydrophobic chains in micelles, are still controversial. In the manuscript, the aggregation pattern of TX100 has been studied using sPREs (solvent Paramagnetic Relaxation Enhancements)-based NMR (Nuclear Magnetic Resonance spectroscopy). It was found that the average positions of the protons in the TX100 micelle are consistent with those in the multilayer staggered spherical micelle model with the p-tertoctylphenyl groups dispersing in the different layers.

## 1. Introduction

**Figure 1:**
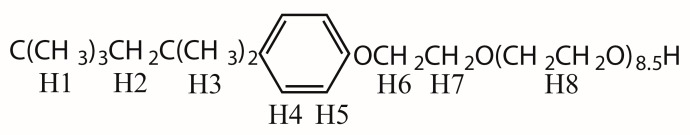
Formula and proton numbering of TX100.

TX100 (Triton X-100, C_14_H_22_O(C_2_H_4_O)_9.5_) is a typical nonionic surfactant that has a hydrophilic polyethylene oxide chain and a hydrophobic hydrocarbon chain with an aromatic group in the middle (Figure 1). The TX100 molecules assemble into micelles in solution at very low concentration, the Critical Micellar Concentration (CMC) of which is 0.2 to 0.3 mM [1,2,3]. It is one of the most widely used detergents that can be used to permeabilize the membranes of living cells, serve as a reservoir to extract proteins or organelles from cell lysate [4,5,6], solubilize or stabilize biomembrane components or proteins [7,8,9,10].

The aggregation characterizations of TX100, which are key properties underlying its application, have been extensively studied [11,12,13,14,15]. It has been generally accepted that the hydrophobic hydrocarbon chain of TX100 is packed in the interior of the micellar core, whereas the hydrophilic polyoxyethylene oxide chains stay outside the micelles. However, the detailed aggregation pattern of TX100, such as the boundary between the polar region and the nonpolar region, and the arrangement of hydrophobic chains in micelles is still controversial. Robert J. Robson et al. calculated the aggregated geometry of TX100 micelle and suggested it may be a oblate or prolate shape with monolayer or multilayer spherical aggregated structure with no obvious nonpolar-polar boundary [11]. H.Z. Yuan et al. revealed that the protons at the end of p-tertoctylphenyl were involved in the formation of the surface of the hydrophobic core by bending back, the hydrophilic polyoxyethylene chain of TX100 is bent and coiled along the surface of the hydrophobic core [16]. P.S. Denkova et al. found that the hydrodynamic radius of TX100 micelle increased linearly from 4 nm to 7 nm with the concentration enhancement and suggested that TX100 may aggregated in a two- or multilayer spherical conformation with staggering between the inner polyoxyethylene chain and the outer octylbenzene moiety [1]. Recently, A. D. Nicola et al. found that TX100 micelles are polydisperse mixtures of spherical, oblate and prolate shaped aggregates by using MD (Molecular Dynamics) simulation [17].

In this manuscript, in order to achieve more details about the aggregation pattern of TX100 inside the micelle, solvent Paramagnetic Relaxation Enhancement (sPRE) combined with the 1D selective ROESY NMR technique was applied. It is well known that NMR methods, especially two-dimensional Nuclear Overhauser Enhancement Spectroscopy (2D NOESY) and Rotating-Frame Overhauser Effect Spectroscopy (ROESY), are effective to study the conformation of molecules by giving rich spatial information. However, the information of distances that NOESY or ROESY provide is in short range less than 6 angstroms and is susceptible to spin-diffusion. In contrast, the paramagnetic relaxation enhancement (PRE) technique provides spatial information of long-range distances, and is hardly affected by spin diffusion. PRE has been widely used to investigate conformations of the hydrocarbon chain [18,19,20] and evaluate location of solutes within micelles [21,22,23,24]. sPRE is a type of PRE based on paramagnetic relaxation enhancements derived from an unbound soluble paramagnetic agent; it provides precise information about the solvent exposure of a spin and is well suited for quantitatively defining the interaction interfaces within a complex or a macromolecule [25,26,27]. We have found that sPRE can be used to study the detailed aggregation mode of CHAPS and distinguish the insertion of the molecular inside the micelle [28]. By using sPRE after choosing the unbound paramagnetic probe to avoid the deficiencies of the bound paramagnetic probe, it was found that the micelle has a multilayer aggregated Spherical Micelle model with polyoxyethylene chain entering the core.

## 2. Results and Discussion

### 2.1. ^1^H sPREs of TX100

**Figure 2:**
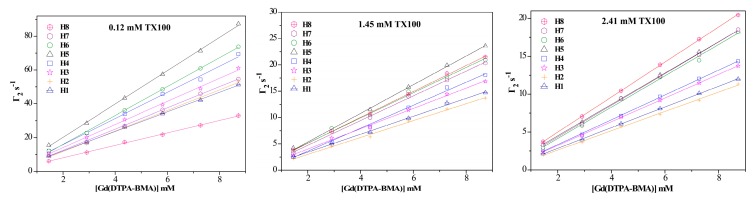
^1^H sPREs of TX100 as a function of the concentration of Gd(DTPA-BMA).

sPREs is the superposition of the PREs on the target nucleus produced by all paramagnetic probes in the solution; it acts as an additional contribution to the normal relaxation rates [29], which is proportional to the concentration of the paramagnetic probe [25]. Figure 2 shows the ^1^H sPREs (Γ_2_) of TX100 upon different concentrations of Gd(DTPA-BMA). Apparently, all Γ_2_s increase linearly with the concentration enhancement of Gd(DTPA-BMA) in great agreement with the sPRE theory. Furthermore, this indicates the absence of specific interaction between the probe and the surfactant, also confirmed by the fact that no significant chemical shift changes are observed upon addition of the paramagnetic probe Gd(DTPA-BMA) (in Figure S1).

**Figure 3:**
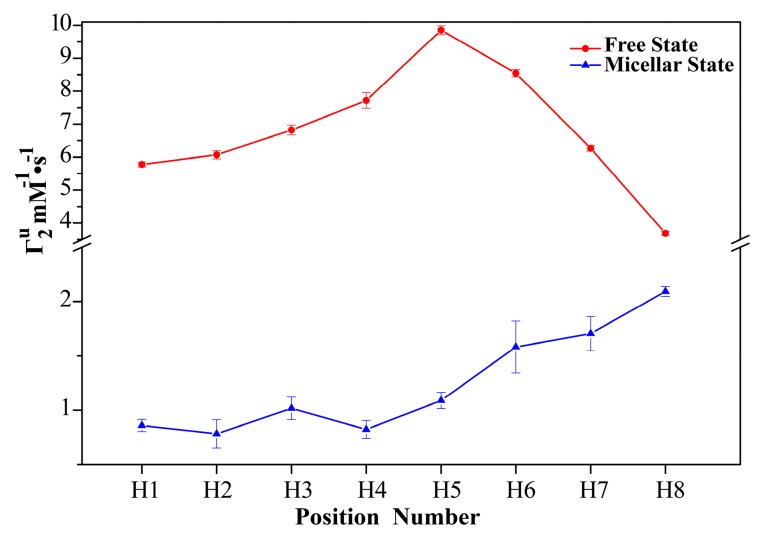
The ^1^H sPREs of TX100 at unit concentration of the paramagnetic probe Gd (DTPA-BMA) in the free and micellar states, respectively.

In the studies of sPRE, the paramagnetic relaxation enhancements produced by paramagnetic probes at unit concentration Γ_2_^u^ are usually used [30]. Γ_2_^u^ is the slope of the Γ_2_ versus the concentration of the paramagnetic probe, which reflects the average insertion depth of each proton in the micelle. Figure 3 shows the ^1^H Γ_2_^u^ of TX100 in the free and micellar states. The Γ_2_^u^ of TX100 in the free state was obtained by linearly fitting of the ^1^H Γ_2_ slope of 0.12 mM TX100, whose concentration is much smaller than the experimental CMC of TX100 (0.29 ± 0.01 mM, in Figure S2). The Γ_2_^u^ of TX100 in the micellar state was achieved by avoiding the sPRE effect from TX100 in the free state [28]. The micelle is a complex system with multiple states, and there are exchanges among them. According to the fast exchange model, the measured apparent Γ_2_^u^ is a population-weighted average of the Γ_2_^u^ of TX100 in free and micellar states. In the manuscript, the Γ_2_^u^ of TX100 in the micellar state was achieved carefully by subtracting the Γ_2_^u^ of TX100 in the free state from the apparent Γ_2_^u^ of TX100 at concentrations above CMC (2.41 mM). 

To extract the insertion depth and evaluate the aggregation pattern of the TX100 carefully, a spherical micelle model has been chosen for the further analysis mentioned below. The shape and size of TX100 micelles are concentration dependent, while, in diluted aqueous TX100 solutions, TX100 micelles are proposed to be more likely to be in a spherical model according to cryo-TEM [31], MD simulation [17], quasi-elastic light scattering [32,33], and PGSE-NMR analysis [1,33]. The size of the micelle is nearly the same under the TX100 concentration of 0.6% *w*/*w* [1] or 2.5% *w*/*w* [32]. In our study, the TX100 concentrations are lower than 0.3% *w*/*w*, the self-diffusion coefficient depends linearly on the inverse concentration of the surfactant, and no other CMCs have been observed (Figure S2), which means no instinct conformational transition or size and shape change of the micelles occurs. As a result, it is reasonable to treat the TX100 micelle as a hard sphere without considering the polydispersity of micelle. 

For a spherical micelle system, since the sPREs of the interested nucleus produced by the solvent paramagnetic probe is the total effect of each paramagnetic center in solution, the Γ_2_^u^ of a special nucleus in the spherical micelle is inversely proportional to cube of the distance of the nucleus to the closest paramagnetic center [25,30,34]. The larger the value of Γ_2_^u^, the shorter the insertion depth of the target nuclei. In this way, the average positions of protons in the micelles can be evaluated. 

In the free state, the Γ_2_^u^s of protons from different positions vary. The Γ_2_^u^s of protons (H4, H5 and H6) in the middle of the TX100 molecule are obviously larger than those of the other protons. This seems to be reasonable because the molecules may fold instead of linearly stretch in the solution to minimize the hydrocarbon/water contact interface. Whereas, in the micellar state, the Γ_2_^u^ s of all protons are much lower than those in the free state, indicating the formation of the micelle. Besides, the Γ_2_^u^s of protons in the micellar state are obviously position related, they become larger and larger with the enhancement of their position number. The Γ_2_^u^s of the protons in the hydrophilic polyoxyethylene end are more than twice as large as those of the protons at the hydrophobic p-tertoctylphenyl end. This result indicates that the conformations of the molecules inside the micelle are different from the molecules in the free state, they may be linearly stretching instead of folding. The Γ_2_^u^s of the protons at the hydrophilic side are relatively higher than that of others, this indicates that the hydrophilic polyoxyethylene moieties are closer to the surface of the micelles, whereas the hydrophobic p-tertoctylbenzene moieties seem to be far from the surface of the micelle because those protons have much smaller paramagnetic effects inside the micelle. It should be noted that the Γ_2_^u^ is also determined by the effective correlation time, but it can qualitatively reflect the relative spatial relationship of each target hydrogen nuclei, since the effective correlation time of Γ_2_^u^ is mainly contributed by the overall motion of molecules [35].

To further evaluate the aggregation pattern of the micelle, the relative sPREs of the other protons to that of H4 (the ratio of Γ_2_^u^s of other studied protons to that of H4) have been calculated by using theoretical model without considering the effective correlation time. 

The PREs are distance sensitive; therefore, in order to obtain the relative sPREs accurately, the hydrodynamic radius of the micelles (4.05 ± 0.05 nm) has been calculated using the Stokes-Einstein equation combining the diffusion coefficients of (4.65 ± 0.01) × 10^−11^ m^2^/s for the micelle and the solvent viscosity of 1.092 cP at 298 K [36]. The diffusion coefficients of TX100 at different concentrations have been measured. To avoid the contribution of TX100 in single molecule state, the diffusion coefficients of the TX100 at the micellar state are derived from the apparent diffusion coefficients at infinitely high concentration, which is the longitudinal intercept in the extrapolation of the diffusion coefficients versus the reciprocal of the concentration above the CMC (Figure S2). Obviously, the radius thus calculated is in excellent agreement with the literature values (from 4 to 4.5 nm) [1,11,32,33]. 

The other distance parameters come from references and the Corey-Pauling-Koltung (CPK) models. The radius of the paramagnetic probe Gd(DTPA-BMA) is 0.35 nm [34]. The length of the octylphenyl group is about 1 nm, while the length of the flexible polyoxyethylene chain for 9.5 polyoxyethylene units in TX-100 micelles is conformation dependent between 3.4 nm (the extended (zigzag) conformation) and 1.6 nm (a random coil conformation) [11]. 

**Figure 4:**
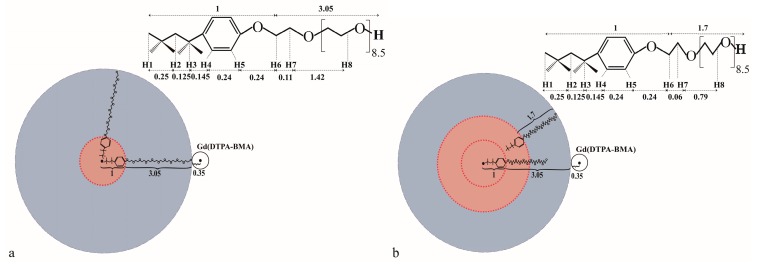
The simplified diagram of the one- (**a**) and two-layer (**b**) aggregated spherical micelle model of TX100; the distances (nm) between some important groups have been marked to clarify the analysis mentioned in the manuscript.

There are two assumed aggregation models for the TX100 micelle: the one-layer model and the multilayer spherical model. In different models, the stretch length of the polyoxyethylene chains inside the micelle has to be different to be consistent with the micelle size, whose hydrodynamic radius is 4.05 nm, and the length of octylphenyl groups is relatively stable at 1 nm, as shown in Figure 4. If the TX100 micelle is in the one layer spherical shape (Figure 4a), the conformation of the polyoxyethylene chain is very similar to the zigzag conformation with the length of 3.05 nm. Whereas, in the multilayer (two layer at least) aggregated spherical micelle model, the conformation of the polyoxyethylene chain should be in the meander conformation, and the length would be 1.7 nm accordingly [11], as shown in Figure 4b. To further illustrate the multilayer model, a three-layer model is constructed by inserting an additional layer in the middle of the two layers of the two-layer model. Obviously, the theoretical apparent Γ_2_^u^ in the multilayer model will be the population-weighted average of the Γ_2_^u^ of TX100 molecules in different layers. 

**Figure 5:**
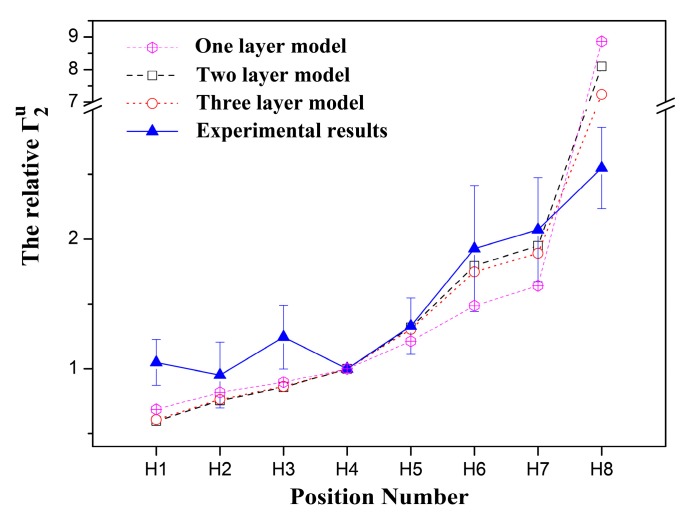
The relative ^1^H Γ_2_^u^ of TX100 of the experimental results in comparison with calculated values for the one-, two- or three-layer aggregated pattern.

Using the distances from the CPK model (Figure 4) and for simplicity, the content of the molecules in each layer is assumed to be nearly the same in the multilayer model, and the theoretical and experimental relative sPREs without considering the effective correlation time are calculated and shown in Figure 5. For the one layer model, the relative sPREs calculated by the insertion depths of all protons (except H4) are apparently inconsistent with the experimental results. The insertion depths of H4, H5, H6 and H7 in the micelles are approximated as 3.53 nm, 3.29 nm and 3.05 nm, respectively. Therefore theoretically, the relative sPREs of H5, H6 and H7 are 1.21, 1.49 and 1.64, respectively, whereas, the corresponding experimental relative sPREs of those protons are 1.33, 1.93 and 2.07, respectively. 

Unlike that in the one-layer model, in the multilayer model, since the size of the micelles are fixed at 4.05 nm, the average distance between the observed protons except H8 and the closest paramagnetic centers will become shorter because a part of the TX100 molecules in the micelle are in the outer layers (Figure 4b). As the sPREs are inversely proportional to the cube of the distance of a specific nucleus to the closest paramagnetic center, the closer the protons are to the surface of the micelle, the more growth of the corresponding Γ_2_^u^s of the protons. Therefore, the relative sPREs of those protons (H5, H6 and H7) would become larger and much closer to the experimental results (Figure 5) in the multilayer model. In the two-layer model, the obtained theoretical relative sPREs of H5, H6 and H7 are 1.32, 1.79 and 1.95, respectively, which are nearly the same as the corresponding measured ones (1.33, 1.93 and 2.07, respectively). 

In the three-layer model, the theoretical relative Γ_2_^u^s of the protons at the polyoxyethylene chains of the TX100 molecules (H5, H6 and H7) are also close to the corresponding experimental ones (Figure 5). However, compared with that in the two-layer model, the theoretical relative Γ_2_^u^ of the proton (H8) at the tail of the TX100 molecules becomes smaller (Figure 5). This is reasonable because the distance between H8 and the closest paramagnetic center is shorter compared with that of H4, the sPREs of H8 decreased faster than that of H4 when some of the TX100 molecules distribute in the middle layer. 

In fact, for the multilayer structure, more molecules should be distributed in the outer layer. Therefore, the theoretical relative Γ_2_^u^s of the protons (H5, H6 and H7) in the multilayer model will become larger and be closer to the corresponding experimental ones. For example, the theoretical relative sPREs of protons (H1 to H3) will be 1.35, 1.88, and 2.05, respectively, if all TX100 molecules are in the outer layer in the two-layer model.

As the hydrocarbon chains of TX100 molecules are flexible and able to bend randomly in the micelle, the actual average insertion depths of the protons (except H4 and H5) will deviate from the theoretical depths according to the above aggregated models. In the one-layer model, although the relative sPREs of the protons (H1, H2, H3, and H8) become closer to the experimental ones as will be described in the multilayer model for the bending, the theoretical results of H6 and H7 will either stay unchanged or deviate farther away from the corresponding experimental results. In addition, the distances between the observed protons (H1, H2 and H3) in the p-tertoctylphenyl moiety and the closest paramagnetic centers are all over 4.00 nm, the maximum distance of the visible sPREs [26], and their actual average positions in the micelles should be closer to the surface of the micelle than the theoretical positions in the one layer model for matching the experimental results. Therefore, it is impossible that TX100 molecules assemble into micelles in the one-layer model with the bending of some hydrocarbon chains. However, in the multilayer model, the bending of polyoxyethylene chains leads to the reduction of the relative sPREs of H8, while bringing it closer to the experimental result. Similarly, the bending of flexible hydrocarbon chains in the p-tertoctylphenyl fragment will decrease the distances between the p-tertoctylphenyl protons (H1, H2 and H3) and H4, which results in the enhancement of the relative sPREs of the corresponding protons, making them get closer to the experimental ones as well. Therefore, it can be concluded that the TX100 aggregates in the multilayer model with the bending of some hydrocarbon chains in the micelle.

It should be pointed out that the relative sPREs may be influenced by the effective correlation time of the observed protons. However, the correlation time of the most protons evaluated by R_1_/R_2_ (in Table S1) are similar, except that of H8 and H1 located at both tail ends of the TX100. These exceptions are reasonable, since the movement of the protons at the ends of the hydrocarbon chains are more flexible and easier to bend randomly in the micelle. 

### 2.2. 1D Selective ROESY of TX100

**Figure 6:**
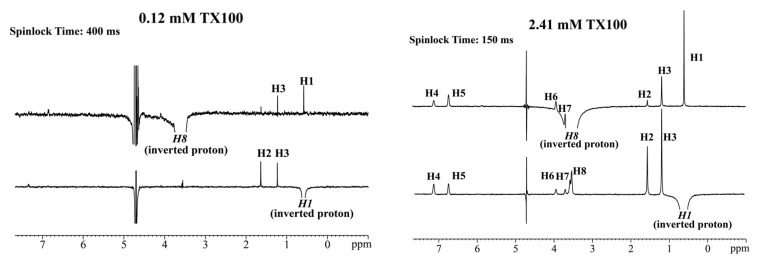
1D selective ROESY spectra of TX100 by selective excitation of H8 and H1.

To further characterize the aggregation pattern of the TX100 micelle, 1D selective ROESY has been applied to study the conformations of TX100 in the free and micellar states. Compared to the two-dimensional NOESY or ROESY experiment, 1D selective ROESY can effectively eliminate spin diffusion and reduce the influence of T_1_ noise [37]. Figure 6 shows the 1D selective ROESY spectra (H8 or H1 is selective inverted) of different concentrations of TX100. Since the CMC of TX100 is 0.29 mM, 0.12 mM and 2.41 mM TX100 are typical samples before and after micelle formation, respectively. In the 1D selective ROESY spectra of 0.12 mM TX100, include the ROEs among vicinity protons (H1, H2 and H3), there are ROEs between H8 and H1 to H3. This result is consistent with the sPRE effect, in which the Γ_2_^u^ of H1, H2, H3 and H8 are all smaller than others, and indicates that the approaching of the nonpolar group and polar group may be induced upon the folding of the polyoxyethylene chain in the dilute solution. 

In the ROESY spectra of 2.41 mM TX100, a lot of ROEs invisible in the non-micelle state, such as the ROEs between H1 and H4 to H7, and the ROEs between H8 and H4, H5 have been observed. Since the molecules of TX100 are linearly stretched in the micelle as indicated by the sPRE results, these results suggest that those characterized ROEs originated mainly from intermolecular interactions between the p-tertoctylphenyl fragment of the outer layer and the first three oxyethylene groups of the inner layer, which is consistent with the multilayer model, and the reported 2D NOESY results [1,16,33]. 

The reliability of those ROESY peaks have been ensured by performing the 1D selective ROESY experiments with different spinlock times, and it was found that the intensities of those ROESY peaks all increase with the enhancement of the mixing time (Figures S3 and S4).

Apparently, the proposed aggregation models based on sPREs and 1D ROSEY are in agreement with the aggregated pattern proposed by Robson and Dennis for intrinsic viscosity measurements [11]. This aggregated pattern with staggered arrangement of the molecules from different layers is reasonable because the oxygen atoms on the polyoxyethylene chain between the two closed layers can be positively charged to interact with the inserted water through hydrogen bond, which stabilizes the two- or multilayer structure by electrostatic interaction between the polyoxyethylene chain from the inner layer and the negatively charged phenyl group from the outer layer.

## 3. Materials and Methods

### 3.1. Instruments and Reagents

D_2_O (99.9%), Triton X-100 (99%), and Gd (DTPA-BMA) (98%) were purchased from Sigma-Aldrich (St. Louis, MO, USA), Nacalal.Tesque (Kyoto, Japan), and LiTTLE-PA Sciences (Wu Han, Hu Bei, China), respectively. All the reagents were utilized without any further purification.

### 3.2. Sample Preparation

D_2_O was used as the solvent for all samples. TX100 at three different concentrations (2.41 mM, 1.45 mM and 0.12 mM) was prepared, configured and titrated with paramagnetic probe Gd(DTPA-BMA). In the self-diffusion coefficient experiments, a high concentration TX100 was prepared first and then diluted to desired low concentrations (a concentration range of 0.1 to 5 mM). To reduce soluble paramagnetic oxygen, the samples were sonicated for 20 min before they were transferred into the 5 mm nuclear magnetic tubes and then stored at ambient temperature and normal pressure overnight before use. In the paramagnetic titration experiment, the titrated concentration of Gd(DTPA-BMA) ranged from 1.45 to 8.72 mM in an ascending order.

### 3.3. NMR Spectroscopy

The sPRE and ROESY NMR experiments were performed at 298 K on a Bruker AVANCE 600 MHz spectrometer equipped with cryogenic TXI probe. The sPRE was achieved by measuring the ^1^H transversal relaxation times of TX100 using CPMG experiments with watergate for water suppression, 32 K time domain data points and 4 s relaxation delay. In the 1D selective ROESY experiments, H1 and H8 were selectively inverted using 80 ms and 35 ms 180-degree Gaussian pulse, respectively. The spin lock time of 50 ms–150 ms, 256 scans, and the relaxation delay of 2 s were applied on 2.41 mM TX100. The spin lock time of the 100 ms–400 ms, 2560 scans and the relaxation delay of 2 s were applied to 0.12 mM TX100. The self-diffusion coefficient measurements were performed at 298 K on a Bruker AVANCE 500 MHz spectrometer equipped with a cryogenic BBO probe. The diffusion coefficients of TX100 were measured using standard DOSY experiment with water presaturation, 16 K time domain data points, and 2 s relaxation delay.

## 4. Conclusions

The aggregation characterizations of detergents are key properties that influence their application. There are two controversial assumption models for the TX100 micelle. In the manuscript, by using sPRE as a tool to evaluate the relative insertion depth for different protons in the micelle, we found that the TX100 micelle is more likely to be a multilayer spherical micelle with bending p-tertoctylphenyl fragments; this staggered structure with no sharp hydrophilic-hydrophobic boundary was further proved by the 1D selective ROESY 

## Supplementary Materials

The following are available online, Table S1: The ^1^H R_1_/R_2_ of TX100 in free and micellar states, Table S2: The ^1^H sPREs of TX100 in free and micellar states, Table S3: The ^1^H insertion depth used for sPREs prediction, Table S4: the relative ^1^H sPREs of TX100 simulated using theoretical mode in comparison with the experimental results, Figure S1: The ^1^H chemical shifts of TX100 in different concentrations do not change with the increasing concentrations of the paramagnetic probe Gd(DTPA-BMA), Figure S2: The self-diffusion coefficients of TX100 versus its reciprocal concentration in D_2_O at 298 K, Figure S3: 1D selective ROESY spectra of 0.12 mM TX100 with different spinlock time, a and b are the spectra with selective inversion of H8 and H1, respectively, Figure S4: 1D selective ROESY spectra of 2.41 mM TX100 with different spinlock time, a and b are the spectra with selective inversion of H8 and H1, respectively, a and b are spectra with selective inversion of H8 and H1, respectively.
