# Supplementary material for: The Study of the Aggregated Pattern of TX100 Micelle by Using Solvent Paramagnetic Relaxation Enhancements
Source: Molecules. 2019 Apr 26;24(9):1649. doi: 10.3390/molecules24091649 (PMC6539090; doi:10.3390/molecules24091649)
Supplement: Supplementary file 1 [file molecules-24-01649-s001.pdf]

Supporting Information

The study of aggregated pattern of TX100 Micelles by using Solvent Paramagnetic Relaxation Enhancements

Liang Zhang[1,2,3], Xin Chai[1,2], Peng Sun[1], Qinjun Zhu[1], Xu Zhang[1,*], Maili Liu[1,*]

1. State Key Laboratory of Magnetic Resonance and Atomic and Molecular Physics, National Center for Magnetic Resonance in Wuhan, Wuhan Institute of Physics and Mathematics, Chinese Academy of Sciences, Wuhan 430071, China;

2. University of Chinese Academy of Sciences, Beijing 100049, China.

3. School of Physics and Optoelectronic Engineering, Yangtze University, Jingzhou, 430023, China

* Corresponding author, ML: ml.liu@wipm.ac.cn; XZ: zhangxu@wipm.ac.

Table S1. The [1]H R_1/R_2 of TX100 in free and micellar states.

Protons	R_1/R_2	
	**Free state**	**Micellar state**
H1	0.72	0.43
H2	0.50	0.10
H3	0.62	0.19
H4	0.30	0.11
H5	0.29	0.12
H6	0.47	0.14
H7	0.48	0.19
H8	0.68	0.52

Table S2. The [1]H sPREs of TX100 in free and micellar states.

Protons	Γ_2^{u} ($s^{-1}mM^{-1}$)	
	**Free state**	**Micellar state**
H1	5.77± 0.07	0.86± 0.06
H2	6.07 ± 0.12	0.78 ± 0.13
H3	6.82 ± 0.15	1.02± 0.10
H4	7.72 ± 0.24	0.82 ± 0.08
H5	9.85 ± 0.13	1.09 ± 0.07
H6	8.55 ±0.11	1.58± 0.24
H7	6.26± 0.09	1.70 ± 0.16
H8	3.68 ± 0.06	2.09 ± 0.05

Table S3. The ^{1}H insertion depth used for sPREs prediction

Protons	Insertion depth (nm)					
	One layer model	two layer model		three layer model		
	1st layer	1st layer	2nd layer	1st layer	2nd layer	3rd layer
H1	4.05	4.05	2.7	4.05	3.375	2.7
H2	3.8	3.8	2.45	3.8	3.125	2.45
H3	3.675	3.675	2.325	3.675	3	2.325
H4	3.53	3.53	2.18	3.53	2.855	2.18
H5	3.29	3.29	1.94	3.29	2.615	1.94
h6	3.05	3.05	1.7	3.05	2.375	1.7
H7	2.94	2.99	1.64	2.99	2.315	1.64
H8	1.525	2.2	0.85	2.2	1.525	0.85

Table S4. the relative ^{1}H sPREs of TX100 simulated using theoretical mode in comparison with the experimental results.

Protons	The relative ^{1}H sPREs			
	One layer model	Two layer model	Three layer model	Experimental values
H1	0.69	0.60	0.61	1.05
H2	0.82	0.75	0.76	0.95
H3	0.90	0.86	0.86	1.24
H4	1.00	1.00	1.00	1.00
H5	1.21	1.32	1.30	1.33
h6	1.49	1.79	1.75	1.93
H7	1.64	1.95	1.89	2.07
H8	8.86	8.10	7.24	2.55

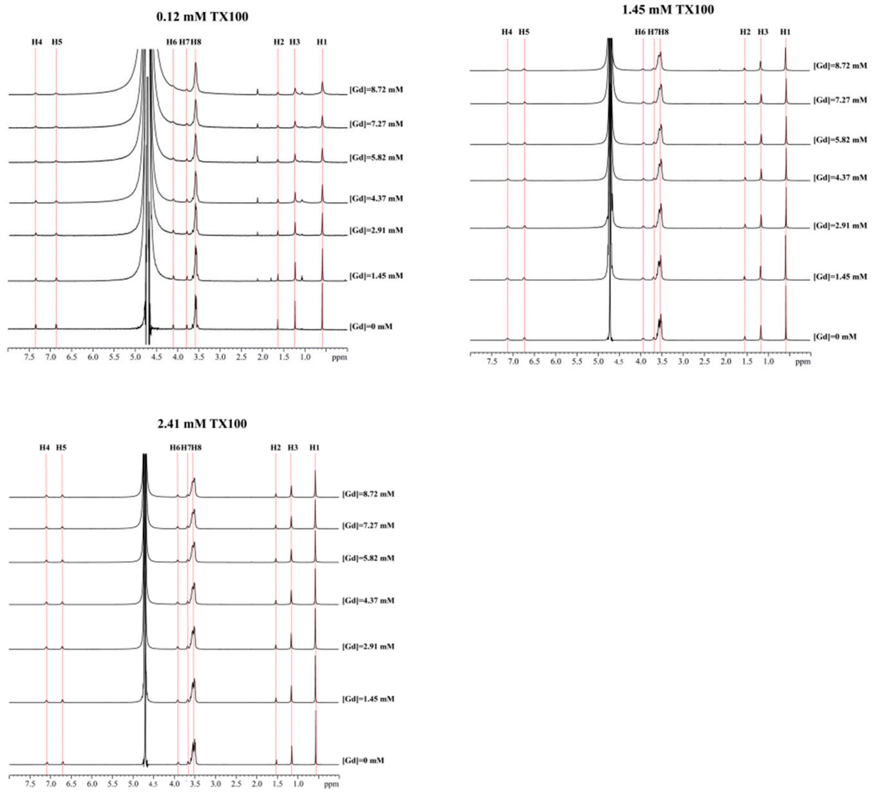

Figure S1. The ^{1}H chemical shifts of TX100 in different concentrations do not change with the increasing concentrations of the paramagnetic probe Gd(DTPA-BMA).

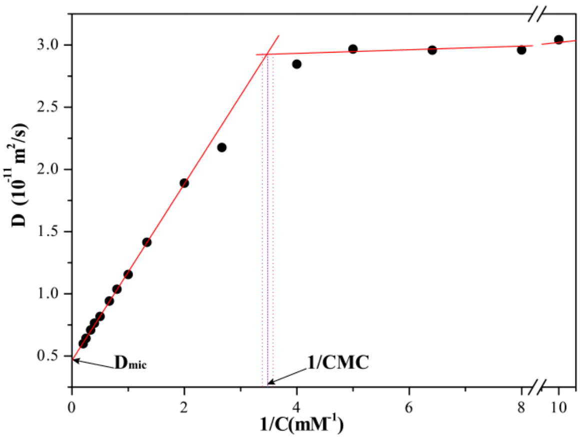

Figure S2. The self-diffusion coefficients of TX100 versus its reciprocal concentration in D_2O at 298 K.

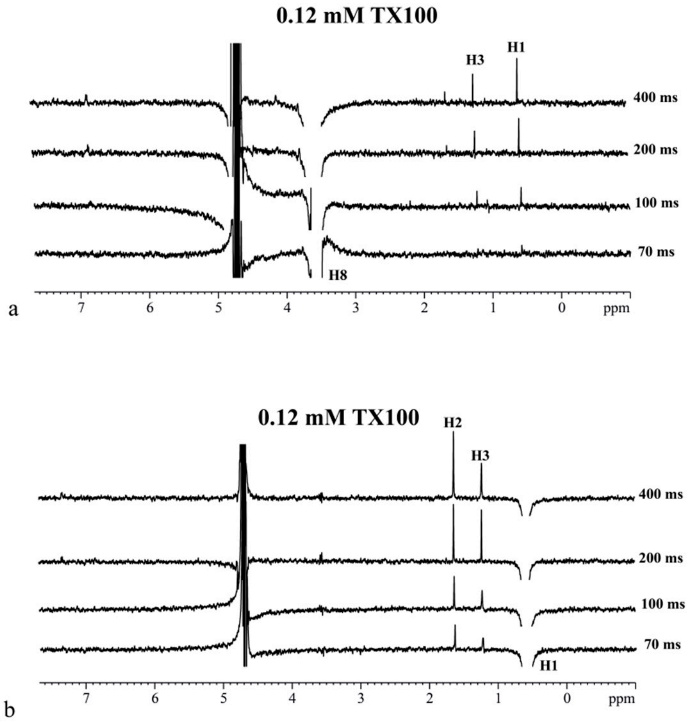

Figure S3. 1D selective ROESY spectra of 0.12 mM TX100 with different spinlock time, a and b are the spectra with selective inversion of H8 and H1, respectively.

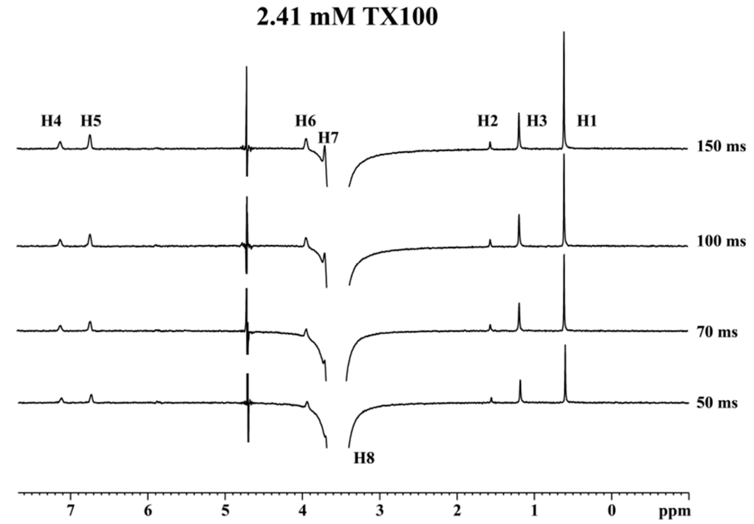

a

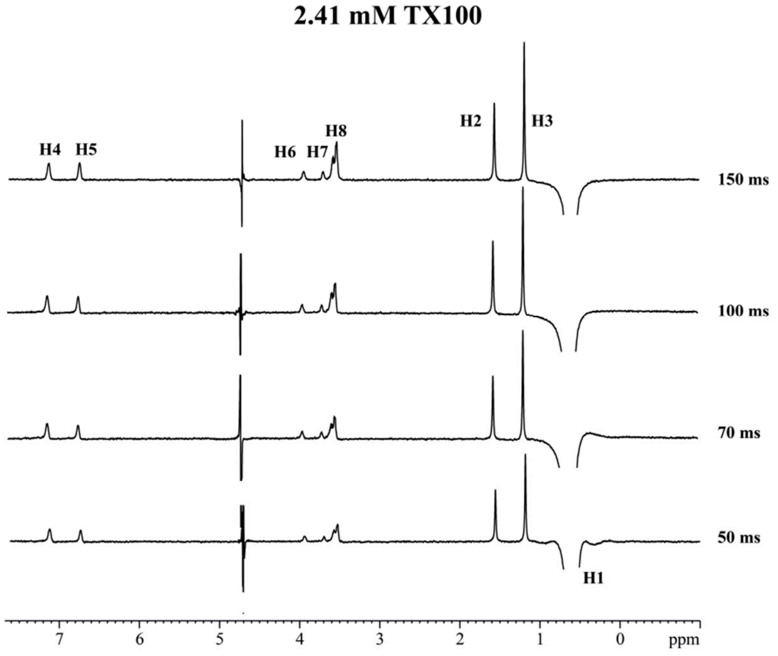

b

Figure S4. 1D selective ROESY spectra of 2.41mM TX100 with different spinlock time, a and b are the spectra with selective inversion of H8 and H1, respectively.
